# Examining personality dimensions in rats using a caregiver questionnaire

**DOI:** 10.1016/j.applanim.2024.106170

**Published:** 2024-02

**Authors:** Holly Brooks, Molly Davidson, Michael Mendl, Vikki Neville

**Affiliations:** Animal Welfare and Behaviour Research Group, Bristol Veterinary School, University of Bristol, Bristol BS40 5DU, UK

**Keywords:** Personality, Pets, Questionnaire, Rats, Welfare

## Abstract

Measures of individual behavioural differences (personality) are highly valuable in many areas of ethology, particularly studies of animal emotion and welfare. However, there are limitations to current behavioural tests of personality. Caregiver questionnaires may provide a complementary approach to overcome some of these limitations and provide a richer insight into personality. Drawing on previous studies, we developed a questionnaire in which caregivers were asked to rate the extent to which a given adjective/term described a rat under their care. We then used factor analysis to identify personality dimensions and assess whether those dimensions mapped to rat sex, rat age, number of companions, predator exposure, or owner experience. We obtained usable data from 296 rat caregivers and identified six personality dimensions: tameness, shyness, liveliness, interspecific sociability, inquisitiveness, aggressiveness. These dimensions are, with the exception of ‘inquisitiveness’, in line with previous studies and the broader literature on personality in non-human animals. With the exception of shyness, which was significantly associated with sex and owner experience, we found no strong evidence for a relationship between the personality dimensions and age, sex, number of companions, predator exposure, or owner experience. Although there remain important issues to be answered by future research, such as whether there is interobserver agreement in personality ratings and the extent to which the personality ratings are repeatable over time, the present study indicates that questionnaires of caregivers could in future provide a valuable tool to assess personality in rats.

## 1 Introduction

Animal personality can be defined as individual behavioural differences that remain consistent over time and context (Budaev, 1997; Reale et al., 2007; Stamps and Groothuis, 2010). Identifying and understanding animal personality is particularly important in studies of emotion in animals (Amendola et al., 2022) where emotions are valenced (positive or negative) states, also known as affective states, that have behavioural, neurophysiological, cognitive and, in humans and likely many other species, conscious components (Mendl et al., 2010; Mendl and Paul, 2020; Paul and Mendl, 2018; Scherer, 2016). This is because the concepts of personality and emotion are closely intertwined. For example, anxiety in humans can be both a ‘trait’ (i.e., relating to personality) and ‘state’ (i.e., more transient) and these are neuroanatomically and functionally distinct in the brain (Saviola et al., 2020). Moreover, different personality characteristics can predispose individuals to experiencing particular emotions, and can influence an individual’s emotional and coping responses to situations that present opportunities or threats (Rusting, 1998; Zelenski et al., 2012). Accordingly, animal personality may moderate the extent to which treatment effects are observed in studies of animal emotion, and hence make interpretation of findings difficult.

There is also increasing emphasis on the role of individual differences in studies of animal welfare (Richter and Hintze, 2019), which largely rely on measures of emotion to make inferences about the impact of particular refinements. For example, there are considerable individual differences in the extent to which playful handling may be perceived as positive by rats (Bombail et al., 2021; Hinchcliffe et al., 2020), and further to this a recent mapping review highlighted that a one-size-fits-all approach is not appropriate in studies of laboratory rat welfare (Neville et al., 2023). Measures of personality are therefore vital to allow us to fully make sense of data and hence make more solid conclusions in studies of animal emotion and welfare.

Rats (specifically Brown rats, *Rattus norvegicus*) are commonly studied in the context of emotion and welfare (Neville et al., 2023), and often subjected to tests including open field, elevated plus maze, and defensive burying tasks from which inferences about personality characteristics such as ‘fearfulness’ and ‘anxiety’ are frequently made (Amendola et al., 2022). However, unless many repeated measures are taken across time, these tests are more likely to be capturing ‘states’, reflecting short-term emotions, rather than ‘traits’, reflecting longer-term stable personality characteristics. Furthermore, behavioural readouts from these tests may be the result of interplay between a number of underlying traits rather than pure measures of a single trait. For example, variance in open field behaviour has been explained in terms of putative underlying ‘boldness’ and ‘activity’ characteristics (Carter et al., 2013; Garcia-Sevilla, 1984; Perals et al., 2017). Behavioural tests of personality thus have limitations and may be unable to effectively identify or disentangle the effects of a range of traits such as ‘activity’, ‘boldness-shyness’, ‘exploration’, ‘sociability’, and ‘aggressiveness’ (Carter et al., 2013; Gartland et al., 2022; Gosling, 2001; Reale et al., 2007) on in-test behaviour. Other methods for assessing specific personality characteristics are thus likely to be valuable.

In companion species, like dogs and cats, questionnaires are frequently used to assess personality (Examples include: Bennett et al., 2017; Hsu and Serpell, 2003; Ley et al., 2008; Salonen et al., 2023; Wiener and Haskell, 2016). While these approaches have limitations too, such as the need to make assumptions that caregivers will interpret and respond to the questions consistently. They are useful because they capitalise on the typically considerable amounts of time that caregivers spend observing and interacting with animals − distilling that experience into quantitative data, and are additionally low cost and easy to implement. Questionnaires may therefore provide a complementary approach to investigate personality in animals, providing that the individuals have sufficient experience with the particular animal being rated (Gosling, 2001, 2002).

We know of just one study of rat personality using owner questionnaires (Korpela, 2011). Owners were asked to rate the extent to which a given adjective/term described a rat under their care. Factor analyses were used to identify personality dimensions, with three dimensions emerging: activity, tameness, and anxiousness. This approach offers promise for future studies of personality in rats, and here we extend the work in order to: (1) assess the reliability of questionnaire-based methods for examining personality in rats by examining whether similar personality dimensions emerge using a similar methodology to Korpela (2011) in a separate study population; (2) assess whether variation in these dimensions might be explained by environmental as well as biological factors. To achieve this, we first developed a questionnaire which was distributed to caregivers. This asked questions about a rat’s environment, before asking about the extent to which adjectives/terms relating to potential personality traits were applicable to the rat. Personality dimensions were then identified using factor analysis, before links between these dimensions and environment were explored.

## 2 Methods

### 2.1 Survey development

We first collated adjectives/terms describing personality traits across both the human and non-human literature, specifically those given in Feaver et al. (1986); Goldberg (1990); Korpela (2011); and Ley et al., (2008). This list of adjectives/terms was then condensed by removing exact duplicates/synonyms and removing those that we jointly decided were not relevant to rats or were too ambiguous (e.g., ‘gullible’, and ‘cruel’). Further to this, we piloted a draft version of the survey with three participants and asked those participants (each of whom had experience owning or working with rats) to suggest adjectives/terms to be removed or added. These suggestions were taken into consideration when finalising the survey. Pilot participants were not eligible to complete the final survey.

### 2.2 Final survey

The final survey contained 73 questions (see Supplementary Material for full survey) and was implemented in Microsoft Forms. The survey was distributed online: via email to rat owners known by the authors, and via social media (Facebook and Twitter), and it was live for a period of two weeks commencing 27th July 2022.

The survey started with a section containing questions relating to the participant (gender, age, and time having owned/worked with rats). Respondents were then asked to complete the remaining questions about a rat in their care. To reduce the possibility of biases from owners deciding which rat to complete the survey for, we asked owners to choose the rat they currently work with or own whose name or ID number comes first alphabetically or numerically. There was then a section containing questions about the rat whose personality they were rating (sex, age, source, whether the rat lives with other rats, whether they live in close proximity to another species, whether the rat has previously been pregnant). Participants were asked to provide a brief freeform written description of the rat’s personality in this section. After this, there was a final section in which participants were asked to rate how strongly they agreed the following adjectives/term applied to their rat: *aggressive towards people, aggressive towards rats, agile, anxious, attentive, bold, careless, cautious, communicative, confident, creative, curious, determined, devious, docile, dominant, easy going, easy to handle, enthusiastic, equable with people, equable with rats, fearful of people, fearful of rats, food-motivated, friendly, generous, happy, helpful, hyperactive, impulsive, indecisive, independent, intelligent, interested, irritable, jealous, lethargic, lively, obedient, opportunistic, patient, playful with people, playful with rats, predictable, protective, proud, rebellious, selfish, shy, sociable with people, sociable with rats, solitary, stubborn, suspicious, systematic, tame, temperamental, trainable, trusting, unemotional*, and *vocal*. In the interests of brevity, we assumed that participants would understand the meaning of each adjective/term and did not provide a definition. For each of these adjectives/terms, participants were asked to rate how strongly they agreed it applied to a particular rat in their care using a Likert scale with an additional option to allow participants to tell us that it wasn’t applicable to rats or was too ambiguous. The following response options were provided: strongly agree, agree, more or less agree, more or less disagree, disagree, strongly disagree, this term doesn’t apply to rats/is ambiguous. We assumed that by priming participants to think about their rat’s personality using the freeform written description of the rat’s personality, and by explicitly asking participants to rate “*personality* trait words”, the participants would understand that these ratings should be based on characteristics perceived to be a stable property of the rat.

The eligibility criteria for the final survey were that participants: (1) resided in the United Kingdom, (2) were over the age of 18, and (3) had owned or worked with at least two rats (specifically Norway rats, Rattus norvegicus) who were over the age of six months for at least one year. This final criterion was to ensure participants had ample experience with rats, particularly mature rats, to rate the personality adjectives/terms reliably. These criteria were communicated to potential participants at the point of distribution, and participants confirmed that they met these criteria prior to survey completion (see Supplementary Material). We did not use any criteria for the individual rat to reduce the possibility of a survivorship bias. For example, within a laboratory context, an age criterion may have biased the sample towards rats used for breeding or handling training purposes (who may be ‘tamer’), and within the context of ownership, a criterion specifying the duration of ownership or time spent observing an individual rat may have biased the sample towards rats who more readily interact with humans or spend time outside of shelters (who may be ‘bolder’).

### 2.3 Analysis

A factor analysis (FA) was conducted in R (R Core Team, 2021) using the ‘fa’ function within the Psych package (Revelle, 2023b) to reduce the personality trait adjectives/terms to personality dimensions on the basis of the ratings (Budaev, 2010). There were 429 survey responses, of which 296 were included in the final analysis − exclusions were due to missing values (see below). We first excluded 18 personality trait adjectives/terms that were rated as not applying to rats or being ambiguous by more than 5% of respondents: careless, creative, devious, generous, helpful, impulsive, indecisive, jealous, obedient, patient, protective, proud, rebellious, selfish, stubborn, suspicious, systematic, unemotional. Following this, we recoded the “does not apply/is ambiguous” response in the remaining data as missing values and excluded data from participants with missing values (either because they did not respond, or selected the “term doesn’t apply to rats/is ambiguous” option for at least one trait adjective/term). The personality trait rating responses were recoded so that they were numeric, from 1 − strongly disagree to 6 − strongly agree. We excluded seven further personality trait adjectives/terms during initial analyses on the basis of a Kaiser-Meyer-Olkin measure of lower than 0.5 (Hair et al., 2018), or communalities of lower than 0.3 (Pallant, 2010). These were: food-motivated, independent, opportunistic, predictable, trainable, vocal, intelligent. The final dataset included 36 trait adjectives/terms: aggressive towards people, aggressive towards rats, agile, anxious, attentive, bold, cautious, communicative, confident, curious, determined, docile, dominant, easy going, easy to handle, enthusiastic, equable with people, equable with rats, fearful of people, fearful of rats, friendly, happy, hyperactive, interested, irritable, lethargic, lively, playful with people, playful with rats, shy, sociable with people, sociable with rats, solitary, tame, temperamental, and trusting, with ratings for each of these from 296 participants. We assessed the suitability of the data for FA with Bartlett’s Test of Sphericity. The factor analysis used the correlation matrix of these 36 trait adjectives/terms, and the promax rotation method was applied. Parallel analysis (PA) was used to determine the number of factors to include. A factor loading threshold of greater than 0.3 was used, and it was not necessary to remove any additional adjectives/terms on the basis of this threshold. A Heywood case had not occurred in our factor analysis: all communalities were lower than 1 (Cooperman and Waller, 2022).

The FA scores outputted by the ‘fa’ function in the Psych package were then analysed. We assessed the validity of these scores following guidelines on the use of factor analysis in animal behaviour research (Budaev, 2010), which recommends use of determinacy indices as outlined by (Grice, 2001) (see also: Revelle, 2023a, for information on the problem of indeterminacy). In particular, we used the maximum proportion of determinacy, p2 (squared multiple correlation between each factor and the original variables), and minimum correlation between any two factor estimates, 2p2 − 1, (Grice, 2001): a p2 value of >0.5 and a 2p2 − 1 value of >0 are considered adequate. These values are also outputted by the ‘fa’ function. We explored whether the factor scores for each dimension identified depended on rat sex, rat age, number of companions, predator exposure, and owner experience using general linear models. Age and sex were included because these are important modifiers of behaviour (Reale et al., 2007), number of companions and predator exposure were included as these are thought to influence emotional state (Dielenberg, 2001; Neville et al., 2023, 2022), and owner experience was included to account for the possibility that ratings would shift as a result of greater experience (e.g., more confident handling through experience leading to greater tameness). Due to the presence of outlying values and violations of model assumptions, data were fitted to a general linear model by robust methods implemented in the ‘robustbase’ R package (Maechler et al., 2023). Backward elimination was used for model selection. The results of the best-fitting model are presented below and the results of the models containing all variables are provided in Supplementary Material for interested readers.

## 3 Results

Following exclusion of data (see Methods), the final dataset comprised data from 296 participants. The majority (84.4%) of these participants identified as female, with 11.5% identifying as male, 3.4% identifying as other, and 0.7% preferring not to say. The most common age group for the respondents was 22−30 (22−30 − 36.5%; 31−40 − 33.4%; 41−50 − 15.5%; 18−21 − 7.4%; 51−60 − 6.1%; 61−70 − 0.3%; 71+ −0.3%). The respondents were largely very experienced with rats, with 43.6% reporting having owned or worked with rats for more than 48 months (13−24 months − 23.3%; 25−26 months − 15.2%; 37−48 months −10.1%; 12 months or less − 7.8%). Most (62.2%) participants owned a species other than rats, and in the majority of cases (94.6%) at least one of the other species owned was a predator species (dog, cat, or snake).

There was a reasonably even split between male and female rats in the final dataset (Female − 50.7%; Male − 49.0%, No response − 0.3%), and 12% of the female rats had previously been pregnant. The most common age for the rats was 13−24 months, and there were very few rats over the age of 36 months (13−24 months − 46.3%; 7−12 months − 26.4%; 25−36 months − 19.9%; Less than 6 months − 5.7%; More than 36 months − 1.7%). The vast majority of rats were pets (Pets: 97.0%; Other −2.0%; Breeding (at breeding facility) − 0.7%; Pet shop − 0.3%). Only a minority of rats were housed on their own and were most commonly housed with more than four other rats (House with 4+ rats − 37.5%; Housed with 1 other rat − 26.0%; Housed with 2 other rats − 18.2%; Housed with 3 other rats − 15.9%; Housed on own − 2.4%).

**Fig. 1:**
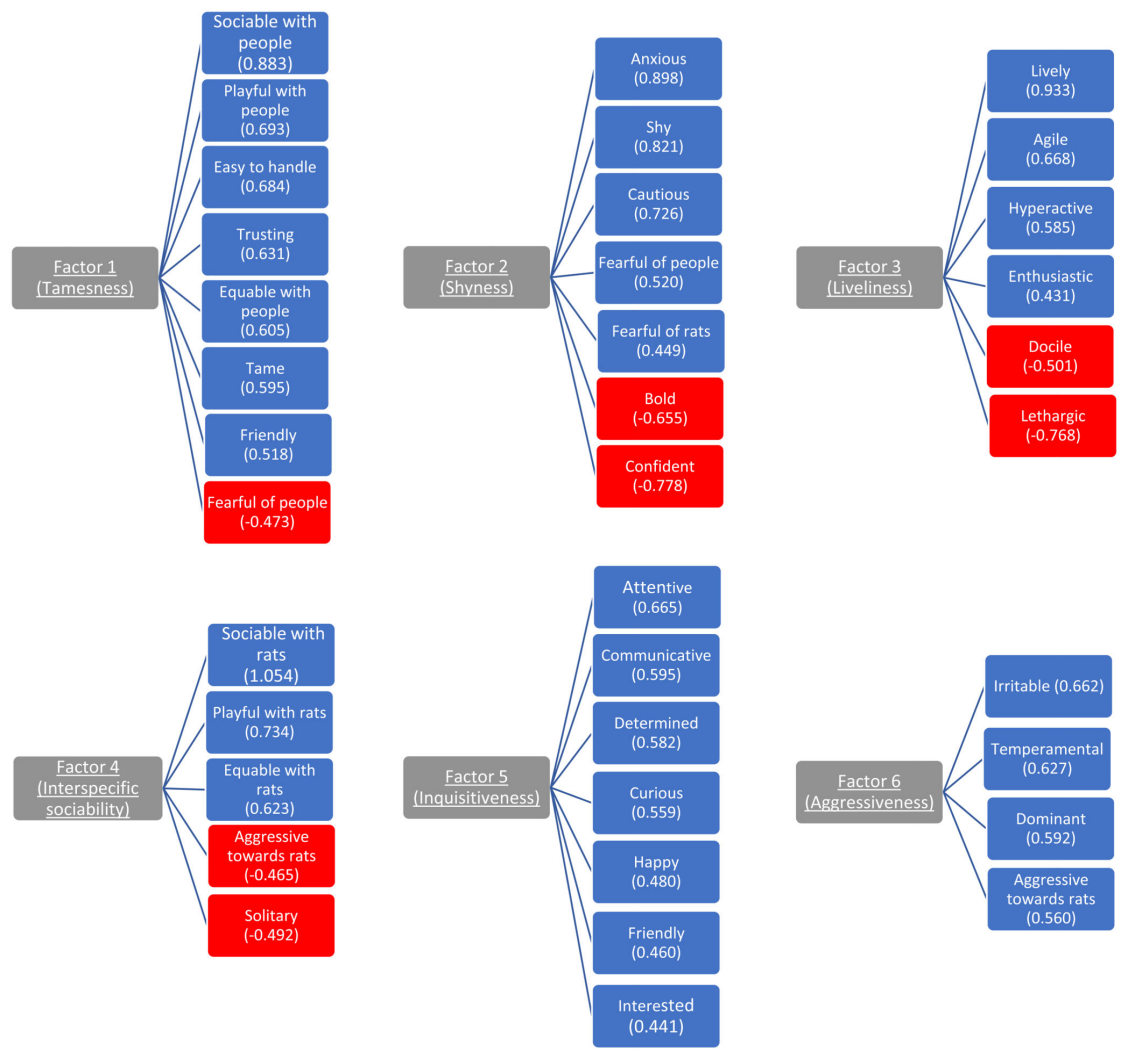
The personality dimensions identified by the factor analysis and the adjectives/terms which load onto these with a loading of >0.4 or <-0.4. The loading values are based on the pattern matrix.

The factor analysis identified six factors. Based on the factor loadings (see Table 1 and Fig. 1), we interpreted Factor 1 to represent *Tameness*: all adjectives/terms which loaded highly onto this factor were semantically related to the term “tame”, “tame” being one of these adjectives. Factor 2 was interpreted to represent *Shyness*: all adjectives/terms were semantically related to ‘boldness’ or ‘shyness’ (commonly used terms to describe personality in the wider literature), with both ‘bold’ and ‘shy’ loading highly, but in opposite directions, onto this factor. Factor 3 was interpreted to represent *Liveliness*: again, all highly loading adjectives/terms were synonyms or antonyms of ‘liveliness’, and liveliness itself was one of the highly loading terms. Factor 4 was interpreted to represent interspecific sociability: this is based on the majority of the adjectives/terms referring to some aspect of social behaviour (with the majority including the modifier, “with rats”). Factor 5 was tentatively interpreted to represent ‘inquisitiveness’ − this term does not fully encapsulate all of the high loading adjectives/terms (for example, ‘happy’), but it directly captures some of the adjectives/terms (for example, ‘curious’, ‘interested’) and it is tangentially related to several others (for example, ‘friendly’ − perhaps reflects being inquisitive about humans; ‘attentive’ and ‘determined’ − perhaps reflect being inquisitive and wanting to investigate or explore various aspects of the environment). Factor 6 was interpreted to represent aggressiveness because ‘aggression towards rats’ was one of the highly loading terms, and also because aggression could stem from the other highly loading adjectives/terms.

Sex (β+/-SE(male)=0.249+/−0.120, z=2.064, p=0.039) and experience (β+/−SE=-0.090+/−0.042, z=−2.126, p=0.034) were found to be significant predictors of the ‘shyness’ scores. An intercept-only model was selected for the following dimensions: tameness, liveliness, sociability, aggression, inquisitiveness, indicating that none of the variables were important predictors of these potential personality dimensions.

**Fig. 2:**
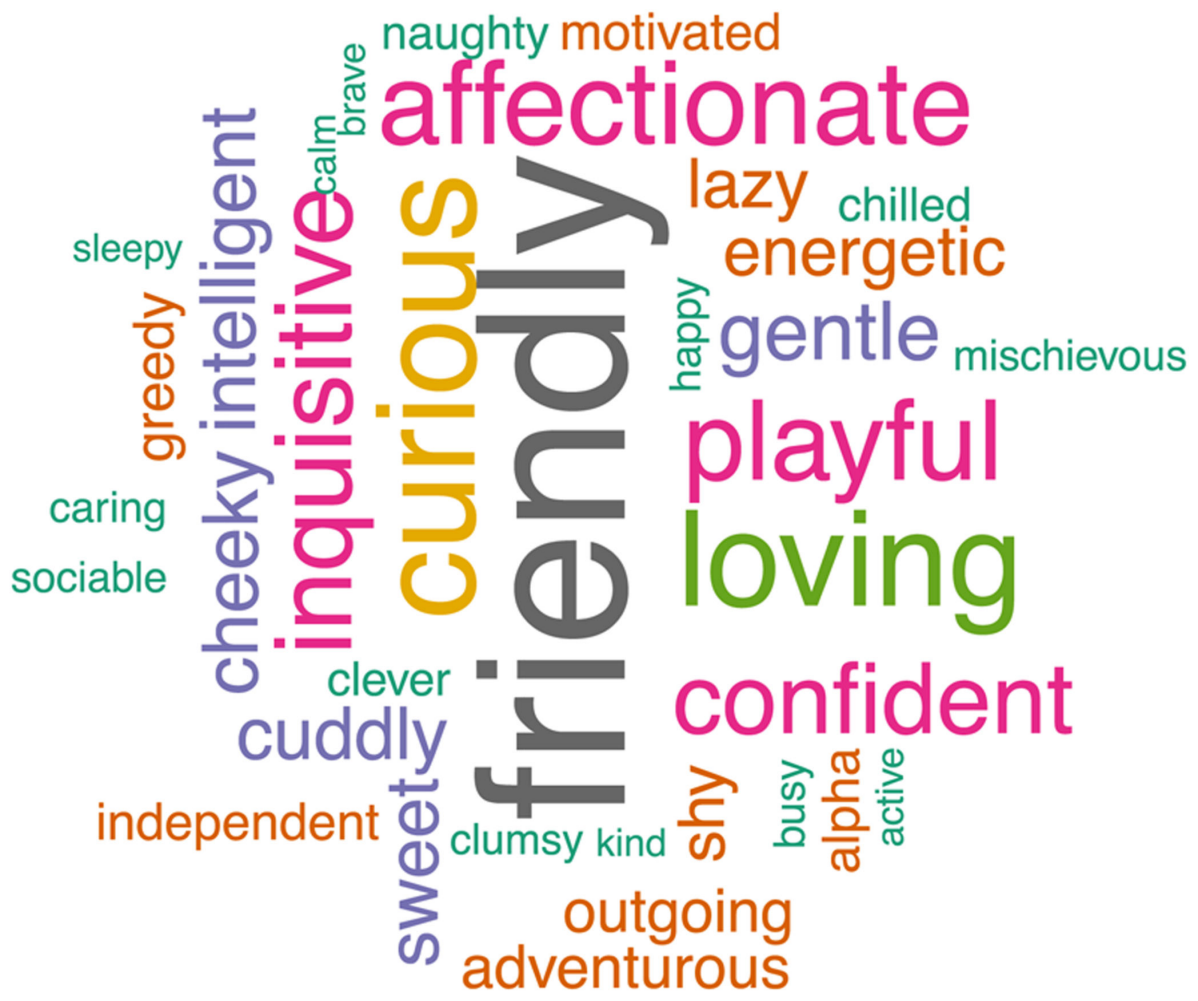
Word cloud of the adjectives caregivers used to describe a specific rat’s personality. Larger font size indicates a higher frequency.

When asked to describe the rat’s personality in the free text question asking caregivers to provide a brief description of a rat’s personality, the caregivers most commonly used adjectives that characterised tameness or social behaviour (e.g., ‘friendly’, ‘affectionate’, ‘loving’, ‘playful’), see Fig. 2. However, other common clusters of words were those related to exploration (e.g., ‘inquisitive’, ‘curious’, ‘adventurous’) and activity (e. g., ‘energetic’, ‘lazy’).

## 4 Discussion

Methods to assess personality are important in studies of animal behaviour, including those investigating emotion. Here, we examined potential personality dimensions in rats, and how these might relate to their environment, using a questionnaire which was distributed to caregivers. We obtained usable data from 296 rat caregivers, and examined these data using factor analysis, leading to the identification of six potential personality dimensions: tameness, shyness, liveliness, interspecific sociability, inquisitiveness, aggressiveness.

The adjectives caregivers commonly used to describe a specific rat’s personality in their free text response prior to providing ratings (i.e., prior to seeing any possible adjectives/terms to describe personality) could be classified according to these dimensions, particularly those relating to tameness, inquisitiveness, and liveliness. This indicates that the properties of ‘tameness’, ‘inquisitiveness’, and ‘liveliness’ are perceived as individual and stable characteristics of rats by those caring for them. However, it’s unclear to what extent this might be a true reflection of personality rather than an interpretation of rat behaviour that is influenced by expectations about and affection towards the rat. Many of the adjectives used are positive rather than being neutral or negative, indicating that the caregiver-rat relationship may have had some sway on the free-text response.

The first three dimensions are consistent with those identified by the Korpela (2011) study of rat personality which identified the following dimensions: activity (which corresponds to our ‘liveliness’ dimension), tameness (as we identified), and anxiousness (which corresponds to our ‘shyness’ dimension). Despite some differences in methodology (e.g. survey development), time (being conducted >10 years apart), and location (with ours conducted in the United Kingdom, vs. in North America and Finland), we were able to derive the same key personality dimensions, hence providing some support for the reliability of questionnaires to assess rat personality.

Our analysis identified three additional dimensions that were not found by the Korpela (2011) study: interspecific sociability, inquisitiveness, and aggressiveness. This may relate to differences in the specific adjectives/terms presented. Firstly, we split several words relating to sociability that were used by Korpela (2011) into two separate words, such as playful (for which we used: “playful with people” and “playful with rats”), sociable (for which we used: “sociable with people” and “sociable with rats”). Additionally, we included “equable with people” and “equable with rats”, and the word “equable” was not included in the Korpela (2011) study. The “with rats” version of these terms loaded highly on the interspecific sociability, and so such terms are likely necessary to provide information about this particular potential personality dimension. Likewise, the majority of the terms which loaded highly onto ‘aggressiveness’ were not included by Korpela (2011): “aggressive towards rats”, “irritable”, and “temperamental” were not included while “dominant” was the only term which was included (although it should be noted that “aggressive towards rats” was initially included in the Korpela 2011 study but was removed due to missing values).

However, the only terms which loaded highly onto “inquisitiveness” in our study that were not included by Korpela (2011) were “communicative” and “happy”, and the remaining five highly loading terms were included in the Korpela (2011) study. It is therefore unclear why the Korpela (2011) study did not identify the ‘inquisitiveness’ dimension. It is possible that this inconsistency between the study outcomes stems from methodological differences in how we decided on the number of factors to include in the factor analysis, this information was not included in the Korpela (2011) study and so we cannot ascertain whether there were indeed differences. It also is possible that this is not a reliable personality dimension in rats, or that it is specific to the particular population of rats whose owners we surveyed.

The dimensions we identified are also in line with the broader literature on personality in non-human animals. The dimensions of “shyness”, “liveliness”, “sociability”, and “aggression” have been widely identified and studied as personality traits in non-human animals (Carter et al., 2013; Gartland et al., 2022; Gosling, 2001; Reale et al., 2007). Activeness, which is comparable to our ‘liveliness’ dimension, has also has been considered to be a non-human analogue of the personality trait of “extraversion” that is commonly described in humans (Garcia-Sevilla, 1984; Gosling, 2001). There is some evidence for the heritability of these traits. For example, in rats, variation in shyness can depend on strain (with rats of the PVG (Piebald-Virol-Glaxo) strain having been characterised as ‘shy’, and rats of the Long Evans strain as ‘bold’) and the neurobiological basis of aggression has been studied (and linked to variation in the expression of brain oxytocin receptors) (Calcagnoli et al., 2014; Naumenko et al., 1989; Veenema and Neumann, 2007).

Although tameness isn’t one of the common personality traits described in the broader literature on animal personality, this is likely to reflect that tameness is largely only applicable to domesticated animals. In studies of personality in companion species, similar personality dimensions have been identified, such as “amicability” in dogs (Ley et al., 2008) and “amiability” in cats (Bennett et al., 2017). Additionally, rats can be selected for levels of tameness towards humans, and these “tame” rats show morphological and neurobiological differences (Albert et al., 2008; Singh et al., 2017), providing evidence that “tameness” may be stable over time and context as per the definition of personality.

The final dimension of inquisitiveness is difficult to interpret because of the low degree of semantic similarity between some of the included personality terms. While “inquisitiveness” links some of the terms (e.g., ‘attentive’, ‘curious’, ‘interested’), its links to others is unclear or tangential at best (e.g. ‘happy’, ‘communicative’). It is also difficult to interpret this dimension in the context of the literature as the adjectives/terms which load highly on this dimension overlap with several previously described personality dimensions. For example, while some terms relate to “exploration” (e.g., ‘curious’, ‘interested’) other terms may relate to the bold-shy (e.g., ‘determined’, ‘happy’) and others to activity (e.g. ‘communicative’). Moreover, the seemingly related adjective of ‘intelligent’ was excluded during data analysis due to low communality. It remains unclear to what extent this dimension might reflect a trait that is stable across time and context in rats, and precisely how it should be interpreted. This could be an avenue for future research.

There was evidence for greater “shyness” in male rat. The reasons for this are not immediately clear; a meta-analysis of personality in non-human animals found no significant sex differences in boldness (Harrison et al., 2022). One possible explanation is that the finding reflects that typical rat housing conditions are more likely to induce anxiety-like states in males than females, which may then be perceived by humans as elevated ‘shyness’ in males. The vast majority of rats in the sample were group housed. Male rats, at least in wild or semi-natural conditions, typically engage in more agonistic social behaviours and are less socially tolerant than female rats (Schweinfurth, 2020). There was also evidence for lower “shyness” in rats cared for by caregivers with greater experience, which might be anticipated if more experienced caregivers are more confident or better at handling and maintaining a good relationship with their rat such that the rat feels more comfortable around them. It would be interesting to explore these weak possible associations in further research.

Otherwise, there was no evidence for a relationship between the potential personality dimensions and rat age, rat sex, number of companions, predator exposure, or owner experience. This is in contrast to the Korpela (2011) study, in which male rats had lower activity scores than female rats. It is possible that there was insufficient power in our study to detect any effects, particular as the Korpela (2011) study obtained a greater sample size. It is also possible that there are no true differences for some or all of these, which might be anticipated if personality traits were largely stable over time and context (although age/sex differences are to be anticipated: (Carter et al., 2013; Gartland et al., 2022; Gosling, 2001; Reale et al., 2007). Indeed, owners may be already taking into consideration factors like age and sex when making ratings; owners might be rating rats according to their prior expectations for a rat of a particular sex or age (e.g., an expectation for older rats to be less lively), such that an older rat might be rated as being highly ‘lively’ because they are livelier than expected for an elderly rat, even if they are in actuality less ‘lively’ than very young rats. This is particularly possible given the level of experience of the owners, and given that the majority of rats were housed with other rats.

Thus, caregiver questionnaires might provide a useful means to study personality in rats, particularly studies undertaken in the fields of affective science and welfare. Our results are not only consistent with the one similar study of personality in rats, but they are also consistent with the wider literature on personality in non-human animals, and there is evidence that some of these personality dimensions may have a genetic basis supporting the idea that they do represent ‘personality’. One important caveat is that the personality ratings ultimately only provide a human perspective on the personality of a particular animal: the ratings will undoubtedly be muddied by their understanding of the adjectives/traits in relation to other humans, and their bond to a particular animal. Similarly, as outlined in the Methods section, we placed no restrictions on how long a caregiver had owned/worked with the particular rat they rated. It is therefore possible that some participants did not have sufficient experience of the particular rat’s personality and behaviour to provide a reliable report − although it is also unclear what would constitute ‘sufficient experience’. Another important caveat to this is that while differences in these dimensions may reflect personality, some variation in these dimensions might also be attributable to changes in ‘state’. For example, “tameness” might be moderated by experience with human handlers (Burgdorf, 2001; Okabe et al., 2020). Likewise, activation or arousal which may be reflected by the term “activity” is considered to be one of the key dimensions of affective state by those that advocate a dimensional approach to defining emotion (Mendl et al., 2010; Russell et al., 1989). Ultimately, it will be important to delineate variation in these dimensions that can be attributed to “personality” as opposed to other potential sources of variation such as a temporary mood state or environmental conditions.

Along a similar vein, we have assumed that we are measuring “traits” because owners will have experience of rats over long time period, and hope they are providing a summary of that in their ratings rather than how the rat is behaving at particular timepoint. Nonetheless, in future studies, it will be important to ask owners to take measures over multiple timepoints to assess stability of measures. It would also be important to assess whether two caregivers for the same rat give ratings that are in agreement (Gosling, 2002). Without this validation, and indeed also assessments of how reliably the subjective ratings made by caregivers links to the rats’ observable behaviour, we cannot be certain that the dimensions extracted here capture rat personality − they are simply potential candidates for dimensions of personality in rats.

Finally, as with many studies of questionnaires, there may be a participation bias such that only enthusiastic owners take part in the study − this could have some bearing on the generalisability of our results. Our attempts to recruit rat caregivers in a range of contexts, such as laboratory technicians, pet shop workers, and pet owners, were largely unsuccessful and our sample largely comprised pet rat owners. Thus, another avenue for future research is to re-attempt to engage caregivers across a variety of contexts so that the generalisability of the results can be assessed. This might be possible by increasing the time the survey is live, and recruiting participants through more relevant channels (e.g., professional bodies for animal technologists).

## 5 Conclusion

In sum, while important issues remain to be answered by future research, the present study indicates that questionnaires of caregivers could provide a useful means to assess personality in rats pending further validation. This questionnaire could eventually provide valuable information to accompany those obtained from studies of rat emotion and welfare, that would enhance the interpretability of data from such studies.

## Supplementary Material

Supplementary Material

## Figures and Tables

**Table 1:** Results of the factor analysis: factor loadings for each adjective/term, as well as the eigenvalue, explained variance, Cronbach’s alpha, p2, and 2p2-1 value for each factor.

	Factor 1 Playful with People	Factor 2 Shyness	Factor 3 Liveliness	Factor 4 Interspecific sociability	Factor 5 Inquisitiveness	Factor 4 Aggressiveness
Aggressive towards people	-0.36	-0.02	0.02	0.10	-0.08	0.35
Aggressive towards rats	0.19	0.02	0.02	-0.47	0.05	0.56
Agile	-0.09	0.02	0.67	-0.05	0.10	-0.14
Anxious	-0.07	0.90	0.07	0.14	0.04	0.14
Attentive	0.04	0.10	-0.02	-0.14	0.67	-0.02
Bold	-0.07	-0.66	0.13	-0.01	0.24	0.29
Cautious	-0.19	0.73	-0.06	0.02	0.31	0.03
Communicative	0.18	0.10	-0.06	-0.12	0.60	0.05
Confident	0.05	-0.78	0.01	-0.07	0.21	0.15
Curious	0.02	-0.09	0.20	0.05	0.56	0.11
Determined	-0.12	-0.14	0.15	0.00	0.58	0.18
Docile	0.17	0.17	-0.50	0.03	0.21	-0.13
Dominant	-0.01	-0.19	-0.22	0.02	0.17	0.59
Easy going	0.36	-0.11	-0.19	0.01	0.09	-0.35
Easy to handle	0.68	-0.14	-0.17	0.03	-0.06	0.01
Enthusiastic	0.19	-0.14	0.43	0.01	0.23	-0.08
Equable with people	0.61	-0.04	0.05	-0.03	0.06	-0.09
Equable with rats	-0.02	0.02	-0.10	0.62	0.01	-0.27
Fearful of people	-0.45	0.52	-0.02	0.06	0.18	0.10
Fearful of rats	0.23	0.45	0.14	-0.30	-0.09	0.12
Friendly	0.52	0.14	-0.12	0.09	0.46	-0.02
Happy	0.01	-0.05	0.00	0.17	0.48	-0.15
Hyperactive	0.12	0.11	0.59	0.09	0.02	0.16
Interested	-0.05	0.03	0.21	0.14	0.44	-0.12
Irritable	-0.02	0.07	-0.08	-0.05	-0.08	0.66
Lethargic	0.05	-0.01	-0.77	0.09	-0.05	0.30
Lively	0.08	0.00	0.93	-0.03	0.03	-0.12
Playful with people	0.69	0.03	0.23	0.25	0.01	0.20
Playful with rats	0.18	0.16	0.14	0.73	-0.03	0.12
Shy	-0.16	0.82	0.00	0.06	0.06	-0.09
Sociable with people	0.88	0.01	0.15	0.09	-0.06	0.09
Sociable with rats	0.12	0.13	-0.10	1.05	-0.10	0.12
Solitary	-0.11	0.11	-0.06	-0.49	0.17	0.16
Tame	0.60	-0.07	-0.06	0.05	0.14	-0.04
Temperamental	-0.14	0.09	0.01	0.07	-0.06	0.63
Trusting	0.63	-0.18	-0.01	0.03	0.03	-0.02
**Eigenvalue**	4.60	3.93	3.11	2.81	2.70	2.38
**Explained variance**	0.13	0.11	0.09	0.09	0.07	0.07
**Cronbach’s alpha (95% Confidence Interval)**	0.90 (0.88−0.92)	0.88 (0.86−0.90)	0.79 (0.75−0.82)	0.82 (0.78−0.85)	0.79 (0.75−0.82)	0.70 (0.64−0.75)
**p2**	0.92	0.93	0.92	0.93	0.85	0.84
**2p2−1**	0.84	0.85	0.83	0.85	0.7	0.69
