# Supplementary material for: Examining personality dimensions in rats using a caregiver questionnaire
Source: Appl Anim Behav Sci. Author manuscript; Available in PMC 2024 Oct 23. (PMC7616731; doi:10.1016/j.applanim.2024.106170)
Supplement: Supplementary Material [file EMS199336-supplement-Supplementary_Material.docx]

**Supplementary Material: Examining personality dimensions in rats using a caregiver questionnaire**

Holly Brooks^1^, Molly Davidson^1^, Michael Mendl^1*,^ Vikki Neville^1*+^

^1^Animal Welfare and Behaviour Research Group, Bristol Veterinary School, University of Bristol, Bristol BS40 5DU, UK

* Equal contribution

+ Corresponding author: vikki.neville@bristol.ac.uk

**A: The survey questions**

**Please confirm you meet the following criteria before beginning the questionnaire.**

Participants must:

- be over 18

- reside within the UK

- currently own or work with rats

- have owned/worked with at least 2 rats over the age of 6 months, for at least 1 year (specifically the species Rattus norvegicus commonly known as Brown, common, or fancy rats - i.e. not pouched rats)

**I confirm that I meet the inclusion criteria for participation in this study.**

Yes

**These questions relate to YOU**

**What is your gender?**

Female

Male

Prefer not to say

Other

**How old are you?**

18-21

22-30

31-40

41-50

51-60

61-70

71+

**How long have you owned/worked with rats?**

12 months or less

13-24 months

25-36 months

37-48 months

More than 48 months

**These questions relate to the RAT**

Please now think about a rat that currently you own or work with whose name or ID number comes first alphabetically or numerically. If the rats you currently own or work with do not have a name or ID number, please just select one rat to think about at random.

**What is the rat’s sex?**

Female

Male

**How old is the rat (at the time of completing this survey)?**

Less than 6 months

7-12 months

13-24 months

25-36 months

More than 36 months

**Which of the following statements apply to the rat?**

I work with this rat in a pet shop

This rat is my pet

This rat is used for breeding (at a breeding facility)

This rat is used in a research context

This rat is used in a context not listed here

**In which context is the rat found (other than those listed previously)?**

Click or tap here to enter text.

**Does the rat live with other rats?**

No

Yes – 1 other rat

Yes – 2 other rats

Yes – 3 other rats

Yes – 4 or more other rats

**Does the rat live in close proximity to other animals (e.g. dog/cat)?**

No

Yes

**If you answered yes to the above question, please specify which other animals live in close proximity to the rat.**

Click or tap here to enter text.

**Has the rat previously been pregnant?**

No

Yes – within the last year

Yes – more than a year ago.

**Please briefly describe the rat’s personality.**

Click or tap here to enter text.

**Rating personality trait words**

Please rate the following personality trait words (61) based on how strongly you feel that they apply to the rat.

|  | Strongly agree | Agree | More or less agree | More or less disagree | Disagree | Strongly disagree | This term doesn't apply to rats/is ambiguous |
| --- | --- | --- | --- | --- | --- | --- | --- |
| Aggressive towards people |  |  |  |  |  |  |  |
| Aggressive towards rats |  |  |  |  |  |  |  |
| Agile |  |  |  |  |  |  |  |
| Anxious |  |  |  |  |  |  |  |
| Attentive |  |  |  |  |  |  |  |
| Bold |  |  |  |  |  |  |  |
| Careless |  |  |  |  |  |  |  |
| Cautious |  |  |  |  |  |  |  |
| Communicative |  |  |  |  |  |  |  |
| Confident |  |  |  |  |  |  |  |
| Creative |  |  |  |  |  |  |  |
| Curious |  |  |  |  |  |  |  |
| Determined |  |  |  |  |  |  |  |
| Devious |  |  |  |  |  |  |  |
| Docile |  |  |  |  |  |  |  |
| Dominant |  |  |  |  |  |  |  |
| Easy going |  |  |  |  |  |  |  |
| Easy to handle |  |  |  |  |  |  |  |
| Enthusiastic |  |  |  |  |  |  |  |
| Equable with people |  |  |  |  |  |  |  |
| Equable with rats |  |  |  |  |  |  |  |
| Fearful of people |  |  |  |  |  |  |  |
| Fearful of rats |  |  |  |  |  |  |  |
| Food-motivated |  |  |  |  |  |  |  |
| Friendly |  |  |  |  |  |  |  |
| Generous |  |  |  |  |  |  |  |
| Happy |  |  |  |  |  |  |  |
| Helpful |  |  |  |  |  |  |  |
| Hyperactive |  |  |  |  |  |  |  |
| Impulsive |  |  |  |  |  |  |  |
| Indecisive |  |  |  |  |  |  |  |
| Independent |  |  |  |  |  |  |  |
| Intelligent |  |  |  |  |  |  |  |
| Interested |  |  |  |  |  |  |  |
| Irritable |  |  |  |  |  |  |  |
| Jealous |  |  |  |  |  |  |  |
| Lethargic |  |  |  |  |  |  |  |
| Lively |  |  |  |  |  |  |  |
| Obedient |  |  |  |  |  |  |  |
| Opportunistic |  |  |  |  |  |  |  |
| Patient |  |  |  |  |  |  |  |
| Playful with people |  |  |  |  |  |  |  |
| Playful with rats |  |  |  |  |  |  |  |
| Predictable |  |  |  |  |  |  |  |
| Protective |  |  |  |  |  |  |  |
| Proud |  |  |  |  |  |  |  |
| Rebellious |  |  |  |  |  |  |  |
| Selfish |  |  |  |  |  |  |  |
| Shy |  |  |  |  |  |  |  |
| Sociable with people |  |  |  |  |  |  |  |
| Sociable with rats |  |  |  |  |  |  |  |
| Solitary |  |  |  |  |  |  |  |
| Stubborn |  |  |  |  |  |  |  |
| Suspicious |  |  |  |  |  |  |  |
| Systematic |  |  |  |  |  |  |  |
| Tame |  |  |  |  |  |  |  |
| Temperamental |  |  |  |  |  |  |  |
| Trainable |  |  |  |  |  |  |  |
| Trusting |  |  |  |  |  |  |  |
| Unemotional |  |  |  |  |  |  |  |
| Vocal |  |  |  |  |  |  |  |

**B: Results of the full robust GLMs investigating the relationship between rate age, number of companions, caregiver experience, predator exposure, and rat sex, on the factor scores for each of the personality dimensions extracted during the factor analysis.**

| **Factor** | **Variable** | **z-Value** | **p-Value** |
| --- | --- | --- | --- |
| F1: Tameness | Age | 0.565 | 0.572 |
| F1: Tameness | Companions | 0.778 | 0.437 |
| F1: Tameness | Experience | 0.603 | 0.547 |
| F1: Tameness | Predator | 1.749 | 0.080 |
| F1: Tameness | Sex | -1.258 | 0.208 |
| F2: Shyness | Age | 0.198 | 0.843 |
| F2: Shyness | Companions | -0.395 | 0.693 |
| F2: Shyness | Experience | -1.997 | 0.046 |
| F2: Shyness | Predator | -1.403 | 0.161 |
| F2: Shyness | Sex | 2.051 | 0.040 |
| F3: Activity | Age | -0.912 | 0.363 |
| F3: Activity | Companions | 0.003 | 0.998 |
| F3: Activity | Experience | -0.692 | 0.489 |
| F3: Activity | Predator | -0.888 | 0.374 |
| F3: Activity | Sex | -1.017 | 0.309 |
| F4: Sociability | Age | 0.656 | 0.512 |
| F4: Sociability | Companions | -0.595 | 0.552 |
| F4: Sociability | Experience | -0.258 | 0.796 |
| F4: Sociability | Predator | -0.786 | 0.432 |
| F4: Sociability | Sex | -1.582 | 0.114 |
| F5: Inquisitiveness | Age | 0.185 | 0.853 |
| F5: Inquisitiveness | Companions | -0.235 | 0.814 |
| F5: Inquisitiveness | Experience | 0.491 | 0.623 |
| F5: Inquisitiveness | Predator | 1.038 | 0.299 |
| F5: Inquisitiveness | Sex | -1.072 | 0.284 |
| F6: Aggression | Age | -0.582 | 0.561 |
| F6: Aggression | Companions | -0.131 | 0.896 |
| F6: Aggression | Experience | -0.558 | 0.577 |
| F6: Aggression | Predator | -0.008 | 0.994 |
| F6: Aggression | Sex | 0.834 | 0.404 |
